# Developing as an early-career clinician academic after a baby

**DOI:** 10.1192/j.eurpsy.2023.197

**Published:** 2023-07-19

**Authors:** O. Kilic

**Affiliations:** Department of Psychiatry, Bezmialem Vakif University Faculty of Medicine, Istanbul, Türkiye

## Abstract

**Abstract:**

Balancing career and family is a hard job for everyone. Academic life with multiple and conflicting demands on our time necessitates prioritizing among responsibilities. This resonates especially for a woman academic who takes care of a baby. The speaker will address specific challenges of mothers who are in both academia and clinical practice, give reflections and elaborate on patterns and ways of dealing with this challenge. The talk is hoped to promote awareness and discussion on addressing the inner and outer sources and planning one’s own way on a career path.

**Disclosure of Interest:**

None Declared

